# Forensic identification of sex determination using lip print analysis in North Bihar, India

**DOI:** 10.6026/973206300210763

**Published:** 2025-04-30

**Authors:** Sumanta Kumar Kolay, Rohit Sharma, Ajit Kumar Chaudhary, Sunil M.K, Swapan Kumar Purkait, Neetha Bhargava

**Affiliations:** 1 Department of Oral Pathology and Microbiology, Government Dental College & Hospital, Nalanda, Bihar, India; 2 Department of Oral and Maxillofacial Pathology & Microbiology, NIMS Dental College & Hospital, Jaipur, NIMS University, Jaipur, Rajasthan, India; 3 Department of Pathology, Madhubani Medical College and Hospital, Madhubani, Bihar, India; 4 Department of Oral Medicine and Radiology, NIMS University, Jaipur, Rajasthan, India; 5 Department of Oral and Maxillofacial Pathology, Buddha Institute of Dental Sciences and Hospital, Patna, Bihar, India; 6 Department of Periodontology, NIMS Dental College & Hospital, Jaipur, NIMS University, Jaipur, Rajasthan, India

**Keywords:** Forensic odontology, lip prints, cheiloscopy, biometric identification, sex determination, pattern analysis, forensic evidence, forensic science, latent prints, digital photography

## Abstract

Forensic identification of sex determination using lip print analysis in North Bihar, India is of interest. The analysis of lip prints consisted of data collection points during baseline, 6 months and 12 months. However, no significant time-related alterations (p > 0.05) were found indicating their enduring nature. Lip print collection through lipstick and digital photography was successful similar to latent method that proved successful except in male participants who used latent documentation at baseline.

## Background:

Forensic science plays a crucial role in justice delivery, law enforcement, and human rights protection. Over time, forensic methods have evolved and been subjected to rigorous scientific validation to ensure their accuracy and reliability. However, the admissibility of forensic techniques in legal proceedings has often been influenced by historical precedents rather than scientific validation [1]. One of the primary challenges in forensic science is the validation of pattern-based evidence, including fingerprints, bite marks, and bloodstains. While fingerprint analysis has long been considered a reliable method for individual identification, its scientific credibility has undergone scrutiny due to concerns regarding its reliability and reproducibility in forensic investigations [2]. Bite mark analysis, in particular, has been a controversial forensic technique due to its inconsistent reliability. Several wrongful convictions have been attributed to misinterpretations of bite mark evidence, raising concerns about its forensic validity [3]. Similarly, despite the unique and permanent nature of fingerprints, their forensic application faces challenges.

Criminals often attempt to evade identification by wearing gloves or altering their fingerprints through surgical procedures. Additionally, conditions such as skin diseases may affect fingerprint recognition, limiting their forensic reliability [4, 5]. In response to these challenges, forensic experts have explored alternative biometric markers such as lip prints, which are unique and unchangeable throughout an individual's lifetime. Lip prints, also known as cheiloscopy, refer to the characteristic patterns of wrinkles and grooves on the labial mucosa. These patterns develop during the sixth week of intrauterine life and remain unchanged, even in the presence of conditions affecting the lips, such as herpes [6, 7]. The uniqueness of lip prints extends even to identical twins, making them a valuable forensic tool for individual identification [8]. Cheiloscopy has gained attention in forensic research due to its potential application in human identification. Various methods have been developed for recording and analyzing lip prints, including the lipstick method, latent lip print method, and digital photography. Despite the growing body of literature on lip prints, further research is required to establish their forensic validity and admissibility in legal proceedings [9, 10]. Therefore, it is of interest to document sex determination using lip print analysis in North Bihar, India.

## Materials and Methods:

This study was conducted at Darbhanga, Bihar over a period of 12 months. The study was approved by the institutional ethical committee, and all participants provided written informed consent.

## Study design:

This was an observational, cross-sectional study designed with comparative analysis to evaluate the efficacy and permanence of lip prints.

## Study sample:

The study population consisted of individuals from the North Bihar region, aged 10-60 years. The participants were selected based on specific inclusion and exclusion criteria.

## Inclusion criteria:

[1] Healthy individuals aged between 10 and 60 years.

[2] Participants with healthy lip mucosa and complete dentition.

## Exclusion criteria:

[1] Individuals with systemic diseases such as diabetes mellitus or diabetes insipidus, which may cause excessive dehydration of the lip mucosa.

[2] Participants with oral mucosal pathologies, lip deformities (*e.g.*, cleft lip), or physical or chemical injuries to the lips.

[3] Individuals with known allergic reactions to lipstick or cellophane tape.

## Sample size calculation:

The sample size was estimated using G*Power (Version 3.1.9.4) with an effect size (d=0.678), a Type I error of 5%, and 95% power (1-β). Based on these calculations, the study required approximately 96 subjects. To account for attrition, the sample size was increased by 20%, resulting in a total of 116 participants (58 males and 58 females).

## Recording techniques:

## Latent lip print method:

Participants were instructed on the procedure for recording latent lip prints. Their lips were cleaned with moist gauze and allowed to dry. They then pressed their lips gently against a glass slab for 3-4 seconds. After the pressure was applied, the glass slab was dusted with black fingerprint powder, and excess powder was removed. The lip print was transferred onto a white bond sheet using 2-inch wide cellophane tape for subsequent analysis.

## Lipstick method:

In this method, a thin layer of dark-colored lipstick was applied to the lips using a disposable applicator. Participants were asked to rub their lips together to ensure an even spread of the lipstick. They were then asked to press their lips gently onto the sticky side of the cellophane tape. The lip print was transferred to a white bond sheet for permanent analysis.

## Digital photography method:

Participants were positioned with their head aligned in the Frankfurt plane, standing at a fixed distance from the camera. The lips were photographed twice using a digital camera (Zoom Lens 14x IS, 5.0-70.0 mm) mounted on a tripod. The camera was placed at a height of 5.5 feet, and the images were captured in natural light. The photographs were transferred to a computer for analysis.

## Classification of lip prints:

The lip prints were divided into four quadrants according to the dental formula, and each quadrant was thoroughly analyzed with the help of a magnifying glass. The prints were classified according to the Suzuki and Tsuchihashi classification system, which categorizes them into six types:

[1] Type I: A clear-cut groove running vertically across the entire lip.

[2] Type I': A partial-length groove of Type I.

[3] Type II: Branched grooves.

[4] Type III: Intersecting grooves.

[5] Type IV: Reticular grooves.

[6] Type V: Other patterns.

For statistical analysis, the lip print types were numbered from 0 to 6, where 0 indicates non-traceable prints, and 1 to 6 represent Types I, I', II, III, IV and V respectively.

## Procedure and follow-up:

Lip prints were recorded at three intervals: at the start of the study (0 months, labeled T1), after 6 months (T2), and after 12 months (T3). These recordings were repeated to assess the permanence and stability of the lip print patterns over time.

## Results:

The study involved 116 participants, comprising 58 males and 58 females, all of whom met the inclusion criteria and consented to participate. Lip prints were recorded using three different methods: the Lipstick Method, the Latent Lip Print Method, and the Digital Method. These recordings were taken at three time points: at the start (T1, 0 months), after 6 months (T2), and after 12 months (T3).

## Lip print recordings at different time intervals:

Statistical analysis was conducted using the one-way ANOVA test to compare the recordings of lip prints across the three methods at T1, T2, and T3. Results showed no significant difference (p > 0.05) in the lip print readings at any of the three intervals for all methods, indicating the stability and permanence of lip prints over time. The data for both males and females revealed that lip prints remain consistent regardless of the recording method used.

## Main values for each method:

## Latent lip print method:

[1] Males: The mean values for T1, T2, and T3 were 1.7586, 1.8793 and 1.8621, respectively, with a standard deviation (SD) of 1.6470, 1.5681, and 1.5833. The p-value was 0.9081, indicating no significant change in the recordings (Table 1).

[2] Females: The mean values were 1.6552 at T1, 2.0351 at T2, and 1.7586 at T3, with a SD of 1.5622, 1.5807 and 1.5818, respectively. The p-value was 0.4116, indicating no significant difference over time (Table 1).

## Lipstick method:

[1] Males: The mean values were 2.3103 at T1, 1.9483 at T2 and 2.0517 at T3, with a SD of 1.2733, 1.2899 and 1.4318. The p-value was 0.7645, suggesting no significant differences (Table 1).

[2] Females: The mean values were 2.069 at T1, 1.8103 at T2 and 1.6897 at T3, with a SD of 1.6422, 1.4444 and 1.3273. The p-value was 0.5434, confirming no significant change (Table 1).

## Digital method:

[1] Males: The mean values were 2.2586 at T1, 2.2456 at T2, and 2.2069 at T3, with a SD of 1.4087, 1.5033, m and 1.4601. The p-value was 0.6543, indicating no significant difference in the lip print recordings (Table 1).

[2] Females: The mean values were 2.0517 at T1, 2.0172 at T2, and 2.0000 at T3, with a SD of 1.5719, 1.5727, and 1.5894. The p-value was 0.4544, confirming no significant variation over the study period (Table 1).

## Statistical comparisons of recordings between time intervals:

Pairwise comparisons using Tukey's ANOVA between the time intervals (T1, T2, and T3) also showed no statistically significant differences, supporting the findings of the permanence of lip prints over time. For instance, for the latent lip print method in males, the differences between T1 and T2 (0.57), T1 and T3 (0.49), and T2 and T3 (0.08) were all not significant, with p-values well above 0.05 (Table 2). Similarly, for females, no significant changes were observed (p > 0.05).

## Comparison of the methods:

The comparison of the three methods revealed that there was no significant difference in lip print recordings between the lipstick method, latent lip print method, and digital method over the three intervals. However, at t1, males showed a significant difference between the latent lip print method and both the lipstick method and digital method, but the latter two methods did not show a statistically significant difference between each other (Table 3).

## Conclusion from statistical analysis:

The results suggest that all three methods of recording lip prints (Latent Lip Print Method, Lipstick Method, and Digital Method) are equally effective in capturing stable and permanent lip print patterns, irrespective of the gender. The study findings reinforce the potential use of lip prints as a reliable and permanent method for individual identification in forensic investigations. These tables summarize the key findings from the statistical analysis of lip print recordings, demonstrating the stability and consistency of lip prints over time and across different recording methods. The absence of significant differences in the readings confirms the permanence of lip prints as reliable forensic evidence.

## Discussion:

In forensic science, identification of individuals plays a crucial role in solving crimes, especially when conventional methods like fingerprinting are not available. Among the various alternatives, lip prints (also known as cheiloscopy) have gained recognition due to their permanence and uniqueness, making them potentially useful for identifying individuals [1]. However, the forensic application of lip prints has not been extensively studied, and there is still a lack of consensus on the reliability and efficacy of different recording methods. The present study aimed to compare three common techniques for recording lip prints-lipstick method, latent lip print method, and digital photography method-and evaluate their stability and potential for sex determination. The results of this study demonstrated that lip prints remain stable over time, as evidenced by the lack of significant differences in recordings made at three different time intervals (0 months, 6 months, and 12 months) for both males and females using the three methods [2, 3]. Similar findings have been reported by other researchers who have studied the permanence of lip prints over different periods [4, 5]. Kapoor *et al.* found that lip print patterns, particularly Type I and Type III, show significant sex differences and remain stable over six months, making them useful for forensic identification in the Marathi population [6].

Moreover, Badiye *et al.* (2013) identified significant sex-based differences in lip print patterns in a Central Indian (Marathi) population, with Type II predominant in males and Type IV in females, supporting their forensic applicability [7]. Regarding the efficacy of the recording methods, this study found no significant differences in the quality or accuracy of the lip prints recorded by the three techniques. The lipstick method, latent lip print method, and digital photography method all produced consistent results across the time intervals, suggesting that each method can be used effectively to record lip prints for forensic purposes [8, 9]. This is consistent with previous studies that have compared different methods for recording lip prints. For example, latent lip print and lipstick methods yielded similar results when used for sex determination [10, 11]. In contrast, some studies have suggested that the digital photography method might be superior, as it allows for better detail and clarity in capturing lip print patterns [12, 13]. Despite the comparable efficacy of the methods, the study also explored the potential of lip prints for sex determination. While earlier studies have reported sexual dimorphism in lip prints, the present study did not find significant differences between male and female lip prints using the three methods [14, 15]. Atreya *et al.* (2022) observed that lip prints exhibited distinct gender differences in the Bihar population, which contrasts with the findings of this study [16]. These discrepancies could be attributed to the variations in sample size, geographical location, and demographic factors. Furthermore, some researchers have questioned the reliability of lip prints for sex determination due to the high degree of individual variation [17]. In addition to sex determination, the study also explored the limitations of lip print analysis. The small sample size (116 subjects) and the confined geographical area of the study (North Bihar) may have influenced the findings, and broader, multi-regional studies are needed to confirm the applicability of lip print analysis across diverse populations [18]. Moreover, variations in lip print patterns due to ethnic differences, as well as the choice of surfaces for latent print collection, can affect the quality of results [19]. The lack of inclusion of pediatric and geriatric populations, as well as individuals from other vulnerable groups such as transgender individuals, is another limitation of this study. Lip prints can be a helpful tool in gender identification, but their accuracy may vary with different recording methods and over time [20]. Further research in these areas is necessary to establish the broader applicability and limitations of lip print analysis [21].

## Conclusion:

Lip prints are a reliable tool for forensic identification due to their unique and permanent nature. All three recording methods-lipstick, latent, and digital photography proved equally effective. It should be noted that further studies with digital advancements may enhance accuracy.

## Figures and Tables

**Table 1:** Lip print recordings by all three methods at three different time intervals (T1, T2, T3)

**Time Interval**	**Latent Lip Print Method (Males)**	**Latent Lip Print Method (Females)**	**Lipstick Method (Males)**	**Lipstick Method (Females)**	**Digital Method (Males)**	**Digital Method (Females)**
T1 (0 months)	Mean: 1.7586, SD: 1.6470	Mean: 1.6552, SD: 1.5622	Mean: 2.3103, SD: 1.2733	Mean: 2.069, SD: 1.6422	Mean: 2.2586, SD: 1.4087	Mean: 2.0517, SD: 1.5719
T2 (6 months)	Mean: 1.8793, SD: 1.5681	Mean: 2.0351, SD: 1.5807	Mean: 1.9483, SD: 1.2899	Mean: 1.8103, SD: 1.4444	Mean: 2.2456, SD: 1.5033	Mean: 2.0172, SD: 1.5727
T3 (12 months)	Mean: 1.8621, SD: 1.5833	Mean: 1.7586, SD: 1.5818	Mean: 2.0517, SD: 1.4318	Mean: 1.6897, SD: 1.3273	Mean: 2.2069, SD: 1.4601	Mean: 2.0000, SD: 1.5894
P Value	0.9081	0.4116	0.7645	0.5434	0.6543	0.4544

**Table 2:** Pairwise comparisons of lip print recordings between T1, T2, and T3

**Method**	**Males (p-value)**	**Females (p-value)**
Latent Lip Print Method	T1:T2: p = 0.91309	T1:T2: p = 0.39981
	T1:T3: p = 0.93531	T1:T3: p = 0.93382
	T2:T3: p = 0.99815	T2:T3: p = 0.61407
Lipstick Method	T1:T2: p = 0.31192	T1:T2: p = 0.61375
	T1:T3: p = 0.55004	T1:T3: p = 0.35210
	T2:T3: p = 0.90843	T2:T3: p = 0.89899
Digital Method	T1:T2: p = 0.99874	T1:T2: p = 0.99239
	T1:T3: p = 0.98021	T1:T3: p = 0.98299
	T2:T3: p = 0.98886	T2:T3: p = 0.99810

**Table 3:** Comparison of lip prints recorded by three methods at T1, T2, and T3

**Method**	**T1 (Mean ± SD)**	**T2 (Mean ± SD)**	**T3 (Mean ± SD)**	**P Value**
Latent Lip Print Method (Males)	2.31 ± 1.27	2.04 ± 1.58	1.86 ± 1.58	0.0148
Lipstick Method (Males)	2.31 ± 1.27	1.95 ± 1.29	2.05 ± 1.43	0.031
Digital Method (Males)	2.26 ± 1.41	2.25 ± 1.50	2.21 ± 1.46	0.4678
Latent Lip Print Method (Females)	1.76 ± 1.65	1.88 ± 1.57	1.76 ± 1.58	0.3117
Lipstick Method (Females)	2.07 ± 1.64	1.81 ± 1.44	1.69 ± 1.33	0.3289
Digital Method (Females)	2.05 ± 1.57	2.02 ± 1.57	2.00 ± 1.59	0.6371
